# Growth and Slaughter Characteristics of Weaning Male Kids of Turkish Native Goat Breeds

**DOI:** 10.3390/ani11102788

**Published:** 2021-09-24

**Authors:** Uğur ŞEN, Emre ŞİRİN, Ayşe Gül FİLİK, Hasan ÖNDER, Dariusz PIWCZYŃSKI, Magdalena KOLENDA

**Affiliations:** 1 Department of Agricultural Biotechnology, Faculty of Agriculture, Ondokuz Mayıs University, Samsun 55139, Turkey; 2 Department of Agricultural Biotechnology, Faculty of Agriculture, Kırşehir Ahi Evran University, Kırşehir 40100, Turkey; emre.sirin@ahievran.edu.tr (E.Ş.); aysegulcivaner@ahievran.edu.tr (A.G.F.); 3 Department of Animal Science, Faculty of Agriculture, Ondokuz Mayıs University, Samsun 55139, Turkey; honder@omu.edu.tr; 4 Department of Animal Biotechnology and Genetic, Faculty of Animal Breeding and Biology, Bydgoszcz University of Science and Technology, 85-796 Bydgoszcz, Poland; darekp@pbs.edu.pl (D.P.); kolenda@pbs.edu.pl (M.K.)

**Keywords:** indigenous breeds, meat production, non-carcass parts, organ weight, weaning

## Abstract

**Simple Summary:**

The carcass characteristics of Turkish native goat breeds are affected by the rearing conditions after weaning. This study aimed to compare the growth, slaughter, and carcass characteristics of male kids from Angora, Hair, Honamlı, and Kilis breeds at weaning age to eliminate the impact of rearing conditions. Honamlı kids had higher birth weight, daily weight gain, carcass weights (hot–cold), and yields (hot–cold) than those of other breeds (*p* < 0.05). Therefore, the Honamlı breed could be used as a fattening material genotype due to its superior carcass characteristics compared to other Turkish native breeds.

**Abstract:**

There is little knowledge about the carcass potential of Turkish native goat breeds raised under different rearing conditions. It is necessary to compare the carcass characteristics of these breeds to minimize the effect of the rearing conditions. Therefore, this study aims to compare the growth, slaughter, and carcass characteristics of male kids at weaning age to eliminate the impact of rearing conditions. Kids born to Angora (*n* = 6), Hair (*n* = 6), Honamlı (*n* = 6), and Kilis (*n* = 6) Turkish native goat breeds, ranging in age from 2–3 years, were slaughtered at 90 days of weaning age and carcass characteristics were determined immediately. There were differences (*p* < 0.05) among breeds in terms of birth weight (BW) and daily weight gain (DWG) from birth to weaning age. Honamlı kids had higher BW and DWG than those of other breeds (*p* < 0.05). Similarly, carcass weights (hot–cold) and yields (hot–cold) of Honamlı kids were higher compared to kids born to other breeds (*p* < 0.05). Additionally, Honamlı and Hair kids had higher longissimus-dorsi (LD), and semitendinosus (ST) muscle weights compared to Kilis and Angora kids. Interestingly, Kilis kids had lower BW and DWG than those of other breeds (*p* < 0.05). There were significant differences (*p* < 0.05) among breeds in terms of non-carcass parts and organ weights. Positive correlations were calculated between cross-sectional area and weight (r = 0.793; *p* < 0.01), length and weight (r = 0.723; *p* < 0.01), and depth and weight (r = 0.698; *p* < 0.01) in LD muscle of all kids. A similar correlation trend was calculated for the ST muscle (cross-sectional area and weight; r = 0.699; *p* < 0.01, length and weight; r = 0.751; *p* < 0.01, and depth and weight; r = 0.528; *p* < 0.05) in all kids. In conclusion, the present study results showed that Honamlı kids could be used for fattening material due to their good carcass quality compared to other native breeds.

## 1. Introduction

Goat breeding is a traditional animal production branch that is generally carried out in underdeveloped and developing countries [1]. All over the world, goats are raised in non-preferred areas such as mountainous, heath, stony, or rocky land areas, which cannot sustain agricultural activities, and are an essential source of livelihood and food for low-income farmers [2]. The studies conducted in developing countries, in which the number of goats is high, revealed that goat farming has a social aspect besides its economic part [2,3,4].

According to the data of Food and Agriculture Organization (FAO) for 2019 [5], there were approximately one billion goats in the world. The top five countries with the highest number of goats are India (13.6%), China (12.5%), Nigeria (7.5%), Pakistan (5.6%), and Bangladesh (6.5%). Turkey ranks first among European countries in terms of goat population, with its goat assets exceeding 11 million heads, representing approximately 47% of the European continent’s goat inventory [5,6]. Additionally, Turkey has a 1.02% share in terms of goat assets and is among the top 20 countries in the world [5]. Turkey is an essential country in terms of live animal population and animal genetic resource diversity among European countries.

Goats have material and moral importance in the Anatolian culture and played an essential role in the historical process in the spiritual and economic factors in Turkish people’s nutrition, clothing, and shelter [7]. Various native goat breeds, which are bred in different geographical regions and are adapted to those conditions, are raised in Turkey [8]. In addition, goat farming is carried out within farming systems, including village herds or transhumance or nomadic herds in mountainous and forest or less-favored areas, which are not suitable for crop production and other livestock species practices [9,10]. Turkey has thirteen national and local native goat breeds named Hair, Angora, Norduz, Honamlı, Kilis, Damascus, Gürcü, Ispir, Kaçkar, Abaza, Malta, and Akkeçi. While Angora, Hair, Honamlı, and Kilis breeds constitute a large proportion of Turkey’s goat population (92%), other local and exotic breeds account for a meager share [8,10,11].

Although Turkey is among the leading countries in the world in terms of goat assets, almost all of the goat population consists of local breeds with low yield potential, but good adaptation to different climatic conditions [8,10,11]. Goat farming is primarily carried out according to the extensive breeding system, with little or no additional feeding and general way of life instead of at a commercial scale in Turkey and many developing countries [9,10]. Moreover, kids of indigenous breeds, raised in different geographical regions of Turkey, are grown similarly until weaning, but growth differences occur according to breeding practices linked to the area’s conditions following weaning [8,10,11]. Kids that will not be used for breeding in traditional goat farming are slaughtered at the age of 4–6 months with a live weight of 10–15 kg [12,13]. Today, consumers primarily in the Mediterranean region prefer the meat of young suckling kids (2–3 months of age with 6–11 kg live weight) because the meat is considered more tender, juicy, and tasty [14,15]. There is scarcely any information about the actual fattening potential of kids born to native breeds to produce goat meat, which prevents the profitable goat farming of farmers. Thus, it is necessary to put forward evaluation criteria that will minimize the effect of the region’s conditions and determine the fattening potential of the native breeds.

Turkey has seven different geographical regions, and goat breeders similarly raise kids until weaning using traditional farming in each region [8,10,11]. In addition, consumers in Turkey and Mediterranean countries prefer weaned kid meat more than beef and lamb [14]. For this reason, determining the carcass characteristics of kids from native breeds at weaning may provide information that will shed light on the future growth potential of these breeds without being affected by the environmental and climatic conditions in those regions. Although there were some studies about fattening performances and carcass characteristics of Angora, Hair, Honamlı, and Kilis breeds and their crossbreeding [16,17,18,19,20,21,22,23] in intensive farming, there are no comparative studies about the slaughter and carcass characteristics at the weaning age of these breeds in traditional farming. Therefore, this study aims to compare the growth, slaughter, and carcass characteristics of weaned kids born from Angora, Hair, Honamlı, and Kilis breeds raised in traditional farming.

## 2. Materials and Methods

All operations of the study were approved by Kırşehir Ahi Evran University, Local Animal Care and Ethics Committee (approval number: 021013-4.1.9). The study was carried out during the breeding season, which covers the mating, gestation, birth, and growth periods of kids until weaning, of goats in Turkey (from September to May). Male kids were obtained from private farms included in the national sheep and goat-breeding project carried out in five different provinces; Ankara (Angora; *n* = 6), Tokat (Hair; *n* = 6), Antalya (Honamlı; *n* = 6), and Kilis (Kilis; *n* = 6). Secondary data consisted of experimental period meteorological data (monthly averages of temperature, rainfall rate, and relative humidity) of study provinces were obtained from the Turkish State Meteorological Service. Monthly averages of the outdoor temperature (°C) rainfall rate (kg/m^2^/month), and relative humidity (%) during the breeding season for the study areas (Ankara, Tokat, Antalya, and Kilis) are presented in Table 1. Male kids of all breeds were selected from does with singletons and during their second birth and were reared according to the traditional system of the regions, i.e., similar feeding and management practices. The does from each breed were vaccinated for all known diseases in their region by the Provincial Directorate of Agriculture. Health control and internal and external parasite treatments of all does were performed before the experimental period. Kids from each breed were born from February to the beginning of March in the same year. Kids were housed in a pen as groups with their mothers until weaning on each private farm. The kids were fed their mothers’ milk until weaning, and according to their appetite of 0.1 kg/day/kid, meadow grass hay was offered after 15 days of age. For the first 15 days after birth, the does were kept in the house with their kids. During this period, does were fed 1.0 kg/day/doe of meadow grass hay. Moreover, water and mineral stone blocks were offered *ad libitum* to both does and kids. After this period, the does were allowed to pasture graze without additional concentrate in extensive areas during the daytime and to suckle their single kid during the nighttime in a barn where they were offered 0.5 kg/day/doe of meadow grass hay. The grazing areas of each breed were natural grassland dominated by *Anndropogon ischoemum*, *Festuca arundinacea*, *Medicago sativa*, *Trifolium pratense*, *Bromus cappadocicus*, *Cynodon dactylon*, *Astragalus* sp., *Capsella bursa*, *Hordeum murinum*, *Amaranthus* sp., and *Circium* sp. species. No kids were taken out of a pen until weaning age.

All kids were weaned at 90 days of age and, after fasting for one day, were weighed and the daily weight gain (DWG) of all kids was calculated from birth to weaning. All kids were transferred for slaughter to the slaughterhouse. Immediately after the slaughter, the head, pelt, four legs, fats (internal fat and kidney fat), and some organs (lung, liver, kidney, heart, spleen, testis, empty small intestine, and empty reticulo-rumen) were removed. At the same time, the weights of the hot carcass, organs, and non-carcass parts were determined. The carcasses of kids were stored at 4 °C for 24 h, and the cold carcass weight at the end of the storage period was determined. Hot and cold dressed yields (HCY and CCY) of all kids were calculated as (carcass weight/fasting live weight) ×100. In addition, the chilling loss (CL) of all kids was calculated as HCY–CCY. Following weighing, a cross-section from the mid-belly of the longissimus-dorsi (LD) and semitendinosus (ST) muscles from the right side of the carcasses was taken using a drawing paper. The cross-sectional area (CSA) of all muscles was determined by a direct grid reading [24], and muscle depth (MD) and length (ML) were determined by a digital caliper.

The statistical analysis was conducted using a completely randomized design for traits. The statistical analyses were performed using the SPSS 17.0 package program (1999, SPSS, Chicago, IL, USA). The optimum sample size was determined by a simple randomize sampling method, and the results showed that six repeats in each group were enough for a trait, which had the maximum variance. The observed power of the test was 92.07%, which shows that the used sample size was adequate for reliable results. To estimate the best fitting model (linear, quadratic, cubic or logarithmic) of the explanatory variable relative to the response variable, curve estimation was used. Linear regression analysis was used to determine the effect of metric measurements on the weight of muscles. Relationships between variable traits were determined with Pearson correlation analysis using a 95% confidence interval. Duncan’s multiple comparison tests tested significant differences between means. Results were computed as the mean ± standard error, and statistical significance was declared at *p* < 0.05.

## 3. Results

The growth and carcass characteristics and relative weight of non-carcass parts and some organs of male kids born to Turkish native goat breeds are shown in Table 2. The birth weight and DWG of Honamlı kids were higher (*p* < 0.05) than those of other breeds. Similarly, Honamlı kids had higher (*p* < 0.05) hot and cold carcass weights and yield compared to kids from other breeds. However, the chilling loss of Kilis and Angora kids was lower (*p* < 0.05) than that of Hair and Honamlı breeds. The relative weight of LD (except for Angora) and ST muscles in Honamlı kids was higher (*p* < 0.05) than those of other breeds. There were no significant differences among breeds regarding kidney fat proportions, but Hair kids’ internal fat (except for Honamlı) and total fat ratios were lower than those of other breeds’ kids (*p* < 0.05). Angora kids had a higher (*p* < 0.05) relative pelt weight, but the relative weights of feet and empty small intestines were lower in Angora kids than in kids from other breeds. Interestingly, the relative weight of the empty reticulo-rumen in Honamlı kids was significantly lower (*p* < 0.05) than that of different breeds. There were no significant differences among breeds in terms of the relative weights of the lung, liver, kidney, and testis, but the relative weights of the heart and spleen (except for Honamlı) in Kilis kids were lower (*p* < 0.05) than those of other breeds’ kids.

The analysis of Pearson’s correlation coefficients for the pooled data for all breeds showed no significant correlations between the carcass characteristics and metric measurements of muscles. However, positive correlations were calculated between the muscle weight and MD (r = 0.698; *p* < 0.01), ML (r = 0.723; *p* < 0.01), and CSA (r = 0.793; *p* < 0.01) for the LD muscle of all kids (Table 3). A similar correlation trend was calculated for the ST muscle, and there were positive correlations between muscle weight and MD (r = 0.528; *p* < 0.05), ML (r = 0.751; *p* < 0.01), and CSA (r = 0.699; *p* < 0.01) in all kids (Table 3).

**Figure 1:**
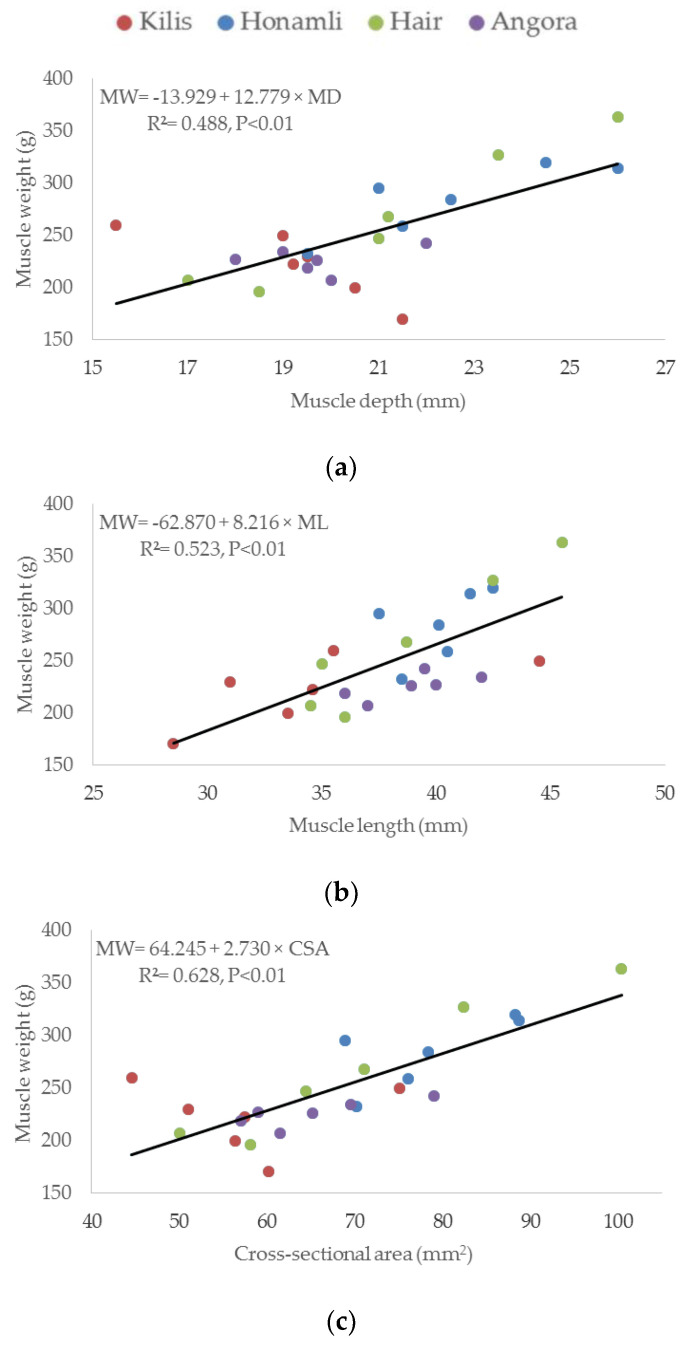
Regression graphs of metric measurements (**a**) muscle depth, (**b**) muscle length, (**c**) cross-sectional area) on weight in longissimus-dorsi muscle for 24 kids born to Turkish native goat breeds.

**Figure 2:**
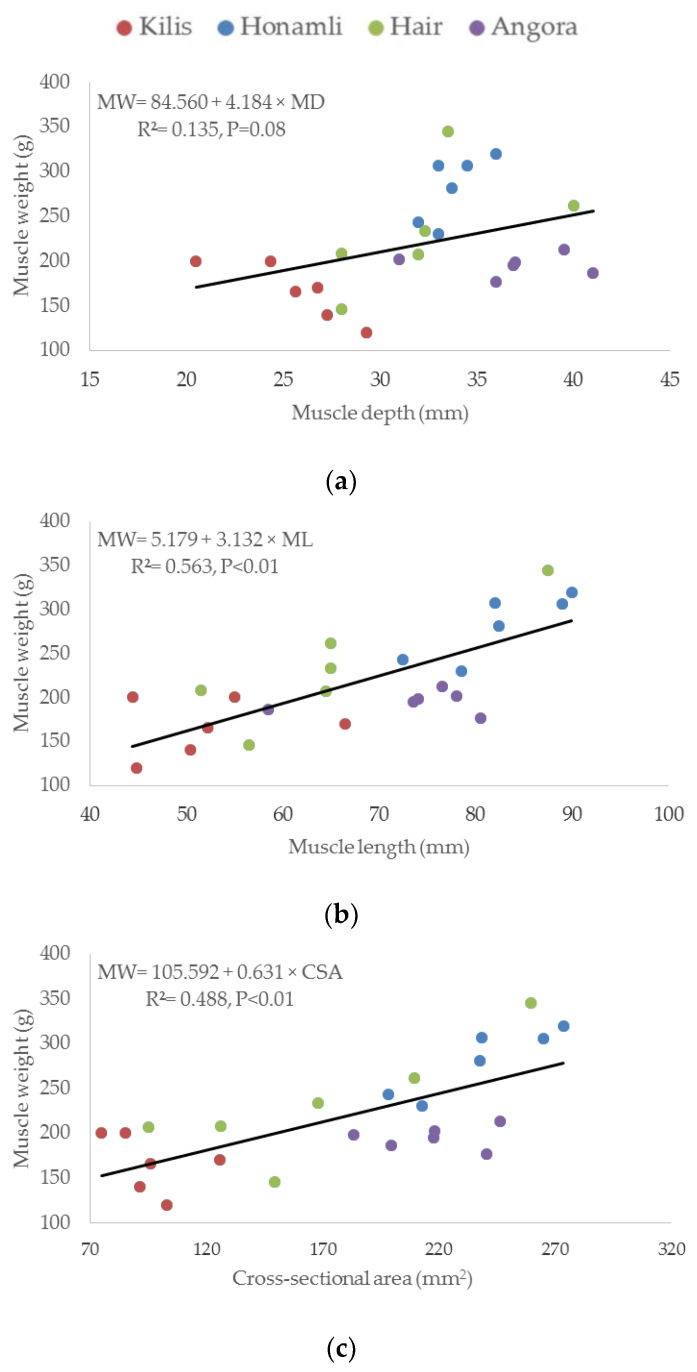
Regression graphs of metric measurements (**a**) muscle depth, (**b**) muscle length, (**c**) cross-sectional area) on weight in semitendinosus muscle for 24 kids born to Turkish native goat breeds.

The results of the curve estimation showed that the shape of all explanatory variables was linear with respect to the response variables. Regression graphs of metric measurements of the weight of LD and ST muscles for 24 kids born to Turkish native goat breeds are presented in Figure 1 and Figure 2, respectively. In the regression analysis, significant relationships were observed between the metric measurements and weights of both muscles. In these models, statistically significant coefficients of determination (R^2^) for MD, ML, and CSA for muscle weight were found as 0.488 (*p* < 0.01), 0.523 (*p* < 0.01), and 0.628 (*p* < 0.01), respectively, for LD muscles (Figure 1). The resulting regression models were as follows: muscle weight = −13.929 + 12.779 × (MD), muscle weight = −62.870 + 8.216 × (ML), and muscle weight = 64.925 + 2.730 × (CSA). According to regression analysis, MD did not affect muscle weight (R ^2^ = 0.135 and *p* = 0.08), but significant relationships were observed in the regression analysis for ST muscle, and these models were statistically significant with a coefficient of determination (R^2^) for ML and CSA of 0.563 (*p* < 0.01) and 0.488 (*p* < 0.01), respectively, for muscle weight (Figure 2). The resulting regression models were as follows: muscle weight = 5.179 + 3.132 × (ML) and muscle weight = 105.592 + 0.631 × (CSA).

## 4. Discussion

Although birth weight is affected by many factors, each Turkish native breed has a characteristic birth weight [10,11]. Birth weight is one of the most critical factors affecting postnatal survival and growth [25,26,27,28]. Previous studies reported that offspring with low birth weight have a lower survival rate and growth rate until weaning [25,26,27,28]. In agreement with past studies [20,21,22], the results of the present study showed that Honamlı kids had a higher birth weight and daily weight gain from birth to weaning than other breeds. Similarly, Akbaş and Saatçi [20], Keskin et al. [21], and Şenyüz [22] reported that Honamlı kids had higher birth weight than Hair, Honamlı × Hair crossbred, and Angora (except for Kilis), and the daily weight gain of Honamlı kids was higher than that of Angora, Hair, and Kilis breeds. Significant differences among breeds were observed for hot and cold carcass weights, in agreement with previous studies [16,17,19,20]. These differences could be attributed to a higher mature body weight and, therefore, higher growth rate and fatness. Honamlı kids, which have the highest adult body weight among Turkish indigenous goat breeds [10], had the highest carcass weight, which led to a higher slaughter weight for finished Honamlı kids following the fattening period. Previous studies reported that weaning weight influenced the post weaning growth performance, and heavy weaned animals reach slaughter weight in a shorter time [29,30]. Additionally, Ekiz et al. [13] and Sañudo et al. [15] also noticed differences among goat breeds considering the differences in mature body size.

Carcass yield is a crucial production criterion, and it expresses the ratio of live weight to carcass weight. Many factors influence carcass yield, such as alimentary tract size and fill, slaughtering procedures, body components (head, organs, intestine, pelt, and feet), body fat distribution, and secondary sex characteristics [31]. In addition, carcass yield must be interpreted carefully, and comparisons must be made between species and breeds [32]. The carcass yield of goats increases with age, body mass, and fatness and ranges from 40 to 56% [31]. Although there are differences in hot and cold carcass yield among breeds, the results of the present study are in agreement with results of previous studies for Angora [23], Hair [19,20], Honamlı [19,20], and Kilis [18] kids. In the present study, the fact that the relative weights of some organs (heart and spleen), gastrointestinal tract components (empty small intestine and reticulo rumen), non-carcass parts (internal fat, pelt, and feet), and the carcass weight differed between breeds explains the reason for the difference in carcass yield. Previous studies indicated that differences in terms of carcass yield among breeds were associated with differences in the weights of non-carcass parts and organs [31,32,33,34]. Interestingly, although the non-carcass parts and organs of Honamlı kids were not lighter than those of other breeds, the carcass yield was higher than that of other breeds. The higher carcass yield of Honamlı kids may have been due to a more significant carcass weight [35]. An increase in carcass yield with increasing carcass weight has been noted in Honamlı breeds, in agreement with Peña et al. [12] and Marichal et al. [36].

Many studies have reported the relationships between carcass (such as weight, yield, and chilling loss) and fat (back fat thickness, total fat, and subcutaneous plus intermuscular fat contents) characteristics [20,37]. Okeudoa and Mossb [37] indicated that the fat content of carcass explained the percentage of the total variation in the chilling rate. The same authors showed that chilling loss was negatively correlated with back fat thickness and total fat [37]. Similarly, Akbaş and Saatcı [20] suggested that the higher chilling losses might be associated with lower back fat thickness and compactness values. The present study determined that Honamlı and Hair kids had a higher chilling loss. Interestingly, compared to other breeds, the carcass of Hair kids had lower internal and total fat contents (Table 2). A higher chilling loss in the carcass of Hair kids was probably due to these carcasses’ lower fatness, especially regarding the Angora and Kilis kids. In agreement with the results of previous studies [20,37,38], the low carcass fat of Hair kids may have increased the chilling loss, although this phenomenon was not detected in Honamlı kids.

Previous studies demonstrated that muscling in lambs is associated with post-weaning eye muscle depth, which is affected by the cellular responses, fiber number, and weight of different muscles [26,39]. The increase in the mass muscle weight during growth is the result of the increase in muscle fiber number and cross-sectional area and the increase in muscle length [40]. Moreover, the differentiation in muscle fibers hyperplasia of fetuses during gestation may affect postnatal muscle weight [28]. Although the weight of the LD and ST muscles did not correlate with carcass weight in all breeds, Honamlı kids had heavier LD (except for Angora) and ST muscle weights compared to the other breeds in the present study. These results suggest that hyperplasia of muscle fibers, which increases the number of muscle fibers during fetal growth and development, in the skeletal muscle mass of Honamlı kids may be higher than in that of the other three breeds, and this may be responsible for increasing the muscle weights of Honamlı kids. This interpretation is supported by the results of Şirin [41], who reported that the total muscle fiber numbers of Honamlı kids were higher than those of kids born to Angora and Kilis breeds.

The correlations of live weight and muscle dimensions, especially in LD muscle, are more valuable indicators for the genetic improvement of carcass yield and composition in livestock [42,43]. Muscle dimensions of LD muscle such as muscle width, depth, area, and the thickness of the fat covering on muscle mass are vital traits in the small ruminant industry for determining the carcass characteristics of live animals; therefore, most selection programs include these traits, and scientists and farmers try to improve these [43,44]. Although there was no a meaningful relationships between muscle (LD and ST) weights and carcass weight, strong relationships between metric measurements and weights of both LD and ST muscles from kids born to Angora, Hair, Honamlı, and Kilis goat breeds were determined. The present study results indicated that metric measurements might estimate the weight of each muscle mass in the carcass. Moreover, metric dimensions may be used as a tool to reveal the meat potential in carcasses of kids from native breeds due to the high correlation with mass muscle weight.

## 5. Conclusions

In conclusion, our results confirm that Honamlı kids slaughtered at weaning are superior to other indigenous goat breeds of Turkey in this study with regards to growth performance, carcass, and muscle yield. This suggests that the Honamlı breed would be a good choice to use in a meat production system where goats are fattened beyond weaning age. Therefore, the potential as a meat goat breed of Honamlı lies in birth weight and pre-wean growth. According to consumer demand, differences among breeds, especially carcass characteristics, may help produce alternative kid meat or meat products. To better understand the carcass and meat quality differences among goat breeds, more comparisons among indigenous breeds are needed as well as the evaluation of different criteria (age, weight, and rearing system) to obtain a broad and objective point of view.

## Figures and Tables

**Table 1:** Average outdoor temperature (OT), rainfall rate (RFR), and relative humidity (RH) during the breeding season in the study areas.

Traits	Ankara	Antalya	Kilis	Tokat
OT (°C)	11.77 ± 1.88	18.87 ± 2.03	17.18 ± 2.19	12.79 ± 1.99
RFR (kg/m^2^/month)	43.6 ± 10.1	68.0 ± 21.7	70.8 ± 20.1	44.01 ± 8.49
RH (%)	63.10 ± 4.25	60.67 ± 3.23	56.12 ± 5.14	64.28 ± 3.29

**Table 2:** Growth and carcass characteristics and relative weight of muscles, non-carcass parts, and some organs of male kids born to Turkish native goat kids.

	Angora (*n* = 6)	Hair (*n* = 6)	Honamlı (*n* = 6)	Kilis (*n* = 6)	*p*
Growth characteristics (kg)					
Birth weight	2.95 ± 0.11 ^b^	2.90 ± 0.29 ^b^	3.55 ± 0.30 ^a^	2.66 ± 0.16 ^b^	0.021
DWG	0.12 ± 0.06 ^b^	0.15 ± 0.09 ^b^	0.17 ± 0.09 ^a^	0.13 ± 0.03 ^b^	0.001
Carcass characteristics					
HCW (kg)	5.49 ± 0.29 ^c^	7.01 ± 0.37 ^b^	8.69 ± 0.44 ^a^	6.09 ± 0.13 ^b,c^	0.001
CCW (kg)	5.28 ± 0.29 ^c^	6.79 ± 0.38 ^b^	8.38 ± 0.40 ^a^	5.95 ± 0.12 ^b,c^	0.001
HCY (%)	40.73 ± 1.23 ^b^	43.38 ± 1.78 ^b^	47.52 ± 2.37 ^a^	42.85 ± 1.98 ^b^	0.004
CCY (%)	40.17 ± 1.20 ^b^	41.97 ± 1.69 ^b^	45.91 ± 2.59 ^a^	41.92 ± 1.87 ^b^	0.007
CL (%)	0.56 ± 0.13 ^b^	1.40 ± 0.23 ^a^	1.60 ± 0.24 ^a^	0.92 ± 0.05 ^b^	0.015
Muscle weights (g/100 g BW)					
LD	1.61 ± 0.06 ^a,b^	1.54 ± 0.26 ^b^	1.76 ± 0.07 ^a^	1.57 ± 0.10 ^b^	0.049
ST	1.40 ± 0.06 ^b^	1.35 ± 0.25 ^b^	1.74 ± 0.08 ^a^	1.18 ± 0.11 ^b^	0.047
Non-carcass parts (g/100 g BW)					
Internal fat	0.86 ± 0.07 ^a^	0.43 ± 0.06 ^b^	0.65 ± 0.15 ^a,b^	0.83 ± 0.05 ^a^	0.009
Kidney fat	0.25 ± 0.02	0.24 ± 0.03	0.25 ± 0.07	0.26 ± 0.02	0.776
Total fat	1.11 ± 0.07 ^a^	0.66 ± 0.07 ^b^	0.90 ± 0.22 ^a^	1.08 ± 0.06 ^a^	0.048
Head	6.87 ± 0.04	7.29 ± 0.51	8.00 ± 0.70	7.33 ± 0.28	0.389
Pelt	13.14 ± 0.43 ^a^	9.08 ± 0.65 ^b^	9.38 ± 1.05 ^b^	7.82 ± 0.16 ^b^	0.001
Feet	2.89 ± 0.03 ^b^	3.83 ± 0.42 ^a^	4.46 ± 0.43 ^a^	3.78 ± 0.11 ^a^	0.014
Small intestine	2.89 ± 0.03 ^b^	3.83 ± 0.42 ^a^	4.46 ± 0.43 ^a^	3.78 ± 0.11 ^a^	0.003
Reticulo rumen	3.14 ± 0.11 ^a^	3.88 ± 0.34 ^a^	2.45 ± 0.30 ^b^	3.20 ± 0.06 ^a^	0.004
Organ weight (g/100 g BW)					
Lung	1.71 ± 0.08	1.82 ± 0.17	2.34 ± 0.29	1.83 ± 0.06	0.082
Liver	2.21 ± 0.05	2.35 ± 0.18	2.42 ± 0.14	2.06 ± 0.07	0.214
Kidney	0.52 ± 0.02	0.52 ± 0.04	0.48 ± 0.07	0.51 ± 0.02	0.801
Heart	0.66 ± 0.02 ^a^	0.64 ± 0.04 ^a^	0.63 ± 0.07 ^a^	0.45 ± 0.01 ^b^	0.010
Spleen	0.39 ± 0.04 ^a^	0.35 ± 0.03 ^a^	0.31 ± 0.05 ^a,b^	0.21 ± 0.01 ^b^	0.011
Testis	0.23 ± 0.02	0.26 ± 0.09	0.20 ± 0.03	0.21 ± 0.01	0.177

^a,b,c^ means in rows with different superscripts are significantly different at *p* < 0.05. DWG = daily weight gain from birth to weaning, HCW = hot carcass weight, CCW = cold carcass weight, HCY = hot carcass yield, CCY = cold carcass yield, CL = chilling loss, BW = body weight, LD = longissimus-dorsi muscle, ST = semitendinosus muscle.

**Table 3:** Pearson correlation coefficients between carcass characteristics and muscle metric measurements for the pooled data of Turkish indigenous goat breeds.

Variable	HCW	CCW	HCY	CCY	CL	LDW	STW
LD							
MD	0.281	0.301	0.395	0.412	−0.216	0.698 **	-
ML	0.074	0.084	0.384	0.376	−0.001	0.723 **	-
CSA	0.210	0.226	0.414	0.415	−0.095	0.793 **	-
LDW	0.343	0.367	0.373	0.390	−0.250	-	-
ST							
MD	−0.028	−0.039	0.063	0.023	0.336	-	0.528 *
ML	0.033	0.032	0.090	0.074	0.120	-	0.751 **
CSA	−0.036	−0.034	0.082	0.070	0.088	-	0.699 **
STW	0.265	0.279	0.385	0.388	−0.096	-	-

HCW = hot carcass weight, CCW = cold carcass weight, HCY = hot carcass yield, CCY = cold carcass yield, CL = chilling loss, LDW = longissimus-dorsi muscle weight, STW = semitendinosus muscle weight, MD = muscle depth, ML = muscle length CSA = cross-sectional area. ** *p* < 0.01, * *p* < 0.05.

## Data Availability

To obtain the data, please contact the authors U.S. and E.Ş.
